# Semi-Automatic Classification of Birdsong Elements Using a Linear Support Vector Machine

**DOI:** 10.1371/journal.pone.0092584

**Published:** 2014-03-21

**Authors:** Ryosuke O. Tachibana, Naoya Oosugi, Kazuo Okanoya

**Affiliations:** 1 Department of Life Sciences, Graduate School of Arts & Sciences, The University of Tokyo, Meguro, Tokyo, Japan; 2 Japan Science and Technology Agency, ERATO, Okanoya Emotional Information Project, RIKEN, Wako, Saitama, Japan; Utrecht University, Netherlands

## Abstract

Birdsong provides a unique model for understanding the behavioral and neural bases underlying complex sequential behaviors. However, birdsong analyses require laborious effort to make the data quantitatively analyzable. The previous attempts had succeeded to provide some reduction of human efforts involved in birdsong segment classification. The present study was aimed to further reduce human efforts while increasing classification performance. In the current proposal, a linear-kernel support vector machine was employed to minimize the amount of human-generated label samples for reliable element classification in birdsong, and to enable the classifier to handle highly-dimensional acoustic features while avoiding the over-fitting problem. Bengalese finch's songs in which distinct elements (*i.e.*, syllables) were aligned in a complex sequential pattern were used as a representative test case in the neuroscientific research field. Three evaluations were performed to test (1) algorithm validity and accuracy with exploring appropriate classifier settings, (2) capability to provide accuracy with reducing amount of instruction dataset, and (3) capability in classifying large dataset with minimized manual labeling. The results from the evaluation (1) showed that the algorithm is 99.5% reliable in song syllables classification. This accuracy was indeed maintained in evaluation (2), even when the instruction data classified by human were reduced to one-minute excerpt (corresponding to 300–400 syllables) for classifying two-minute excerpt. The reliability remained comparable, 98.7% accuracy, when a large target dataset of whole day recordings (∼30,000 syllables) was used. Use of a linear-kernel support vector machine showed sufficient accuracies with minimized manually generated instruction data in bird song element classification. The methodology proposed would help reducing laborious processes in birdsong analysis without sacrificing reliability, and therefore can help accelerating behavior and studies using songbirds.

## Introduction

**Figure 1:**
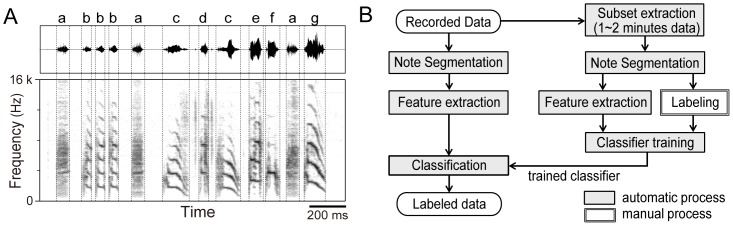
Example of syllable labeling and schematic drawing of the semi-automated labeling procedure. (A) Waveform and spectrogram of a typical Bengalese finch song. Label examples are shown above the waveform panel. Boundaries of syllables and gaps are indicated as dotted vertical lines. (B) Proposed procedure for semi-automated labeling. Stages in gray boxes are automatically processed, and a white box indicates manual processing stage.

Birdsong provides a powerful model for understanding the behavioral and neural bases underlying complex sequential behaviors. Songs of the Bengalese finch (*Lonchura striata* var. *domestica*) consist of successive strings of brief vocalized elements (*i.e.*, syllables) ordered according to complex sequential rules [1]. Therefore, a sequencing system for its song may serve as a unique model for studying systems that generate sequential behaviors, such as human speech. In neuroscientific studies, birdsong analysis usually requires parsing and classifying syllables in continuously recorded data (see Fig. 1A) to explore causal relationships between neural activities and song structures. This processing is highly laborious because the finches vocalize tens of thousands of syllables per day. Moreover, the syllables in finch song exhibit rendition-to-rendition variability, even those appear to have an identical syllable type, on various acoustical features including duration, amplitude, fundamental frequency, and spectral entropy [2]. This study proposes an efficient classification procedure for song analysis using a combination of expert supervision and an automatic classification algorithm.

Several methods have been proposed to replace such laborious processing with an automatic classification system. A template-matching algorithm has been developed that scans over an entire sound recording to identify target elements [3],[4],[5]. An earlier study used dynamic time warping to nullify temporal variation (dilation/compression) within song syllables [3]. That algorithm achieved an accurate classification rate of 97–98% for zebra finch songs using spectrogram templates. Later studies extended the method to utilize a hidden Markov model in which the hidden states represented temporal transitions between acoustical features within a syllable [4],[5]. This extension yielded a higher classification rate of 98–99%. These methods successfully nullified the temporal variation of each element but they did not explicitly handle variation in other acoustical features. The vocalized elements in Bengalese finch songs rarely overlap and should be easily separable in time. Thus, one can employ another approach that uses a simple classification based on multidimensional acoustical features of previously segmented sound units.

We propose a semi-automatic classification procedure using an efficient classifier, the support vector machine (SVM) as one of the supervised learning algorithms. Through this procedure, users perform manual classification on a small population of song elements to generate an instruction dataset by traditional means, such as visual inspection of waveforms and spectrograms. Then, the instruction dataset is used to train a machine classifier that processes the full dataset (see Fig. 1B). To minimize human effort the instruction dataset should be as small as possible, and it is desirable to increase feature dimensions while considering the high dimensionality of acoustical variation in the syllables. In general, a restricted dataset with higher dimensionality increases the risk of over-fitting, which reduces the generalization performance of classifiers. SVM is an effective algorithm that can avoid this problem [6]. For example, the SVM have been widely used to classify songbird species according to their songs in the context of acoustical assessment of ecological environment (e.g. see [7],[8]). In the neuroscientific studies on songbird, successful application of SVM to syllable classification was partially reported in the methods section of Hamaguchi and Mooney (2012) [9]; however, the authors did not fully investigate the capacity of an SVM-based system for song element labeling.

In the present article, we performed two types of validation test. First, we tested the accuracy of classification using segmented syllables of varying size and acoustical features in the training dataset to determine how many samples and what type of feature space were desirable for generating a successful classifier. The first test was performed in an ideal situation, where occurrence rates among syllable types were equalized and highly variable syllables (*i.e.*, introductory syllables) were excluded from the test. Then, we conducted a second test to verify that the proposed method could applied in a real-world situation where nonstationary syllables were included at actual occurrence rates among all syllable types as seen in the original song. Finally, we applied the classification procedure to a large dataset and estimated the classification accuracy to simulate performance under actual usage conditions.

## Materials and Methods

### Recording

Songs of male Bengalese finches (>120 post-hatch day) were recorded using a microphone (Audio-Technica PRO35) suspended above a birdcage inside a soundproof chamber. Each bird was isolated during the recording, and thus all songs were not directed to females. A spatial distance between microphone and bird's head was about 20 cm. The microphone output was amplified using a mixer (Mackie 402-VLZ3) and digitized through an audio interface (Roland UA-1010/UA-55) at 16-bits with a sampling rate of 44.1 kHz. The recordings lasted around 13 hours. The recorded data were down-sampled to 32 kHz. Singing parts in the recordings were automatically detected a succession of eight or more sound elements with gaps lasting less than 300 ms. All birds were derived from an aviary in our laboratory at the University of Tokyo, Japan. The temperature and relative humidity of the aviary were maintained at approximately 25°C and 60%, respectively. The light/dark cycle was 13/11 h.

### Syllable segmentation

Syllables were segmented from continuous song recordings by the following procedure. First, the audio waveform was band-pass filtered at 1–8 kHz, and its amplitude envelope was extracted by full-wave rectification and low-pass filtering at 200 Hz. The amplitude envelope was transformed to a logarithmic scale to obtain the relative sound level. Then, periods containing sounds were detected by setting a sound threshold at the mean background noise level +4 standard deviations (SD). The mean background noise level was detected as a peak in the sound level histogram. The SD was estimated from the full-width-half maximum value of the histogram. This algorithm requires the background noise level to be constant throughout a recording, though the requirement would be satisfied when the recording was performed on each isolated bird in the soundproof chamber, as usual setup for established model animals in neuroscience researches, such as Bengalese finches and zebra finches. Finally, syllables with durations less than 20 ms and gaps with durations less than 3–10 ms were eliminated. A suitable gap threshold was chosen for each bird by visual inspection of the spectrograms.

### Acoustical features

This study tried to utilize high dimensional feature space to cover multidimensional variation in acoustical properties of song syllables. A total of 532 acoustical features were calculated from each sample syllable (Table 1). Spectra, cepstra, and their temporal transitions were included in the feature vector with some additional acoustical features. First, the original waveform of each syllable sample was preprocessed through a high-pass filter (differential filter) to remove baseline biases and to slightly emphasize the higher frequency region. Short-term Fourier transform with a hanning window (FFT size: 256, step: 64) was applied to the waveform generating the spectrogram. The mean spectrum was obtained by averaging the spectrogram across each syllable. The 0-th coefficient of the spectrum (DC component) was not included in the feature vector. The temporal transition of the spectrogram (delta-spectrogram) was calculated by 5-point regression on each frequency band. The absolute values of the delta spectrogram were averaged across each syllable to obtain the mean delta-spectrum (Δspectrum). The set of mean spectrum and Δspectrum is termed ‘Spec’ in this study. Similarly, the cepstrum coefficients and Δcepstrum were calculated for each syllable. Fourier transform and temporal averaging of the spectrogram were performed to obtain the mean cepstrum. The 0th coefficient of cepstrum was also excluded from the feature vector. The temporal transition of cepstrum was calculated for making the Δcepstrum by the same way as the Δspectrum. The set of the mean cepstrum and Δcepstrum features was termed ‘Ceps’.

**Table 1:** Compounded feature set for classification.

feature		dimension
Spec	spectrum	128
	delta spectrum	128
Ceps	cepstrum	128
	delta cepstrum	128
AF	duration	1
	zero cross	1
	feature set ^*1^	9
	delta feature set ^*2^	9
	total	532

^*1.^derived by non-linear transformations of spectrum and cepstrum: spectral centroid, spectral standard deviation, spectral skewness, spectral kurtosis, spectral entropy, spectral slope, peak quefrency, pitch goodness, and amplitude.

^*2.^time derivatives of the nine features (*1).

Additionally, a set of twenty acoustical features, called ‘AF’, was prepared as follows. The first two features were duration and zero-cross. The zero-cross feature was calculated as the number of zero-crossing (from positive to negative) within entire syllable divided by the duration. The feature vector also included nine features that were derived from non-linear transformations of the spectrum and cepstrum: four indices of spectral shape (centroid, standard deviation, skewness, and kurtosis), spectral entropy (or flatness), spectral slope, peak quefrency (or fundamental frequency), size of peak quefrency (or pitch goodness), and amplitude. These features were selected because they could be extracted relatively easily by simple calculation and have been well used in the field of audio feature description [10] (see also [11] for calculation); similar features have been utilized in a well-known song analysis software ‘Sound Analysis Pro’ [12]. Temporal transitions (*i.e.*, delta parameters) of these nine features were also obtained by 5-point regression.

### Classifier setting

A linear SVM that determines classification boundaries by maximizing margins between nearest samples and the boundary hyperplane [6] was used for syllable classification. This study utilized efficient SVM algorithms implemented in a program library written for the MATLAB language (LIBLINEAR ver. 1.93) [13]. The program provided a multiclass classifier consisting of multiple combinations of binary soft-margin SVMs with linear kernel. To test for differences among training algorithms the three types of optimization functions, which were implemented in the program library, were used; L2-regulated L2-loss, L2-regulated L1-loss, and L1-regulated L2-loss functions (termed 2R-2L, 2R-1L, and 1R-2L, respectively). In this study focus was not placed on the theoretical details of these optimization functions (see [13] for the mathematical definitions). The cost parameter of the soft margin (‘c value’) was fixed at 1 after exploring the most suitable value within a range of 2^−15^ to 2^15^. This parameter exploration was performed on the dataset 1 with L = 7, N = 20, ALL condition (see Section 2.5 for dataset description). The classification performance was highest and stable with c value from 10^−2^ to 10^4^.

### Evaluation 1: Exploration of classifier conditions in an ideal situation

In the first evaluation, we tested various classifier parameters to determine the most efficient conditions in an ideal situation where all syllable types had the same occurrence rates. The dataset for the first evaluation (dataset 1) consisted of 64 examples per syllable type for each of eight birds. Syllable types were manually classified by an expert researcher to generate the dataset for classifier evaluation. The 64 samples were randomly selected from the entire set of one-day recordings. Highly variable syllables (*i.e.*, introductory syllables and several noisy syllables) were excluded from the dataset. The number of syllable types ranged from 7 to 11 among the eight birds selected.

The accuracy of the classifier was assessed by cross-validation testing where the training and testing data were chosen from dataset 1 with no overlap. The number of training samples (N) for one syllable label was systematically varied: N = 5, 10, 15, 20, 25, 30, 40, and 50 (8 conditions). The number of test samples was fixed at 10 for each label. The number of label classes (L) was chosen from L = 4, 5, 6, or 7. A set of training and test for each condition (8 sample conditions × 4 label conditions  = 32 conditions) was performed repeatedly on randomly selected labels and samples to eliminate selection biases. For example, when the condition was N = 20, L = 5 for a bird with a maximum of 10 syllable types, 5 syllable types were randomly selected from the possible 10 types. Then, 20 training and 10 test samples were randomly extracted from the pooled dataset of 64 samples. The random selection of labels and samples was repeated 30 and 20 times, respectively. Therefore, the validation was performed 600 times for each condition for each bird. The classification performance was defined as the percentage of correctly classified syllables. Syllable that chance levels differed between conditions with different numbers of labels and can be calculated simply as the inverse of the number of labels (1/L).

Furthermore, we prepared seven different conditions regarding the feature space to explore the influences of feature types on the classification. In the first three conditions, each feature type (Spec, Ceps, or AF) was used separately. Additional three conditions were combinations of the three feature types: Spec+Ceps, Spec+AF, and Ceps+AF. In the last condition all features were used simultaneously (ALL condition). Each acoustical feature in the training data was scaled by z-standardization, which involves subtracting its mean and dividing by its SD. Such scaling was also applied to the testing data using the same scaling factors (mean and SD) as the training data. For the spectrum, Δspectrum, cepstrum, and Δcepstrum features the z-standardization was performed for pooled coefficients but not for each respective coefficient to avoid destroying potential covariance relationships among these coefficients. Additionally, an effect of dimensional reduction on the classification performance was tested by performing the principal component analysis (PCA) on the whole features (ALL condition). The features of training data were z-standardized and underwent PCA. The principal components of test data were calculated using a weight matrix derived for training data. The number of the principal components was varied among 10, 20, 40, 80, 160 and 320.

### Evaluation 2: Application in a realistic situation

The second evaluation was performed in more realistic condition than the first one to verify the applicability of the proposed classification procedure in an actual situation where the syllable occurrence rate was the same as in the original song including highly variable syllables (*i.e.*, introductory syllables and noisy syllables). The dataset consisted of 600∼800 syllables corresponding to 120-seconds of a recorded file for each of the 13 birds (dataset 2, Table 2; see File S1 and S2 for segmentation example, and File S3 and S4 for entire feature matrices). The dataset was generated by randomly collecting song bouts throughout an entire recording while keeping original song sequences in the bouts to preserve the actual occurrence rates. All syllables were inspected and labeled by an expert.

**Table 2:** Number of labels and syllables of dataset 2 and 3.

ID	# labels	Dataset 2, # samples for training					Dataset 3 # syllables
		120 s	90 s	60 s	30 s	15 s	
b01	14	690	507	326	137	46	8610
b06	8	724	548	358	163	70	10750
b09	9	885	637	430	215	112	13129
b10	10	796	604	399	207	101	18767
b11	7	851	648	434	219	105	11580
b13	10	799	607	402	203	99	64825
b14	8	821	620	406	191	84	40836
b16	7	798	598	399	200	100	28633
b17	8	779	592	393	203	107	49013
b18	8	763	578	383	186	91	17399
b19	5	764	580	387	192	97	28893
b20	9	636	498	327	157	74	8581
b21	9	774	594	393	181	80	37709
mean	8.6	773.1	585.5	387.5	188.8	89.7	26055.8
SD	2.1	61.4	44.8	33.1	24.0	18.4	17672.3
Mean syllables/label	-	89.7	68.0	45.0	21.9	10.4	3024.3

Validation was performed as follows. To approximate the total duration of recording data that should be used as the instruction data (labeled manually by the user) to obtain a reliable classifier, the amount of data used for training was varied between 15, 30, 90, and 120 seconds of recording duration. In the 120-s condition 10 samples (corresponding to around 1-second of recording) were used for test and remaining samples were used for training. The actual numbers of syllables used for training are shown in Table 2. Training data were randomly selected from an entire two-minute data, and the remaining samples were used as the test data. The random selection of training data and validation tests were repeated 600 times. Classification performance was evaluated using two indices: correct rates (CR) and Cohen's kappa (*Κ*). CR was simply calculated as correctly classified rates. Cohen's kappa is an unbiased correct rate normalized by the chance level [14] and is defined by the following expression: *Κ* = (CR−c)/(1−c); where c is the chance level. The chance level was defined as the inverse of the number of syllable types for each bird.

In Evaluation 2, all acoustical features were used as the feature space (ALL condition), and the optimization function was fixed at the 2R–2L optimization according to the result from Evaluation 1. Z-standardization of feature vectors was performed in the same way as the previous evaluation but the scaling factors (means and SDs) were calculated from all syllables of pooled data from all birds before starting this evaluation.

### Evaluation 3: Classification of large dataset

The classification procedure was applied to a large dataset to simulate the performance under actual usage conditions. The one-minute data (half of dataset 2) were first used to train the classifier. Then, the trained classifier processed one-whole-day data of 13 birds (dataset 3). The classifier parameters, feature space, and scaling factors were the same as in Evaluation 2. Manual labeling by an expert was performed on a subset of dataset 3 instead of the entire dataset because it included too many syllables (see Table 2). The correct rate for the entire dataset was estimated from the correct rate of the subset. Syllable selection for the subset and the estimation of correct rate were achieved as follows.

First, the classifier was trained using the one-minute instruction dataset. Then, all syllable samples in dataset 3 were labeled by the classifier. Feature vectors of all samples were projected onto evaluation axes; each axis corresponded to a label class, and the projected value represented the likelihood of each sample belonging to each class. Through such projection each sample value was transformed into a normalized space where the class boundary, the margin toward inside of the class, and another side margin were expressed as 0, 1, and -1, respectively. The normalized score can be derived for each label class, and the SVM classifier algorithm used in this experiment judges a data sample as belonging to the class corresponding to the evaluation axis on which the sample shows the highest value (maximum). Here, we defined the evaluation score of each sample as the maximum value among all evaluation axes. The evaluation score should be correlated to the correct rate because a lower score indicates the data sample has a low likelihood of belonging to any label class. Therefore, we extracted the subset data from dataset 3 for each bird at several locations on the evaluation axis within values corresponding to bilateral margins: −0.8, −0.6, −0.4, −0.2, 0, 0.2, 0.4, 0.6, and 0.8. At each location, a maximum of fifty of the nearest samples was chosen within a range of ±0.1 around the location value. Correct rates were calculated for each location and were fitted by a logistic curve as a function of the evaluation score, called the correct rate function. Then, an occurrence probability curve of the evaluation score was derived by averaging proportional occurrence histograms of all birds. Multiplication of the correct rate function and the occurrence probability density provided an estimate of the probability density of correct label. Then, the overall correct rate was estimated by accumulation (or an integral) of the correct label probability density.

### Ethics statement

The experimental procedure and housing conditions were approved by the Institutional Animal Care and Use Committee of the University of Tokyo.

## Results

### Evaluation 1

**Figure 2:**
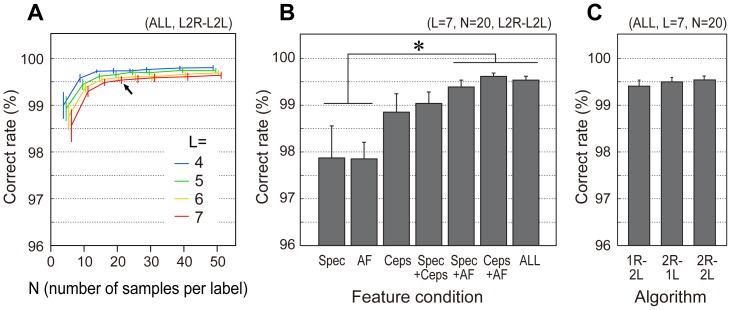
Cross validation performances of classification in ideal dataset situation (Evaluation 1). The correct rates (%) were derived with systematically varying number of labels (L), number of samples per label (N), feature conditions, and optimization algorithms. (A) Correct rates of each of label number conditions (differentiated by line colors) as a function of sample number conditions for ALL-feature and 2R–2L-optimization situation. (B) Correct rates at L = 7, N = 20 location (shown as a black arrow in the leftmost panel) as a function of feature conditions using 2R–2L optimization. (C) Correct rates L = 7, N = 20 location from different optimization functions. Error bars indicate standard error (*n* = 8 birds). *p<0.05 (Tukey-Kramer HSD).

The correct classification rates generally increased with the number of training samples although the rate appeared to plateau at more than 15 samples per label, as evident from the results of the ALL condition using the classifier with 2R–2L optimization (Fig. 2A). The correct classification rates gradually decreased as the number of labels increased from 4 to 7. A training dataset with 20 samples for each label was sufficient to achieve a correct rate around 99.5%. Repeated two-way ANOVA revealed significant main effects for two factors, number of labels (*F*(3,224) = 4.94, *p*<0.01) and number of samples (*F*(7,224) = 22.30, *p*<0.01), but no interaction of them (*F*(21,224) = 0.11, n.s.).

Correct rates for different feature spaces were inspected under L = 7, N = 20 condition (shown as a black arrow in Fig. 2A), and we found highest correct rates in ALL, Spec+Ceps and Spec+AF conditions (Fig. 2B). Repeated one-way ANOVA showed a significant main effect of feature vector (*F*(6,42) = 6.10, p<0.01). Additionally, *ad hoc* multiple comparison between the feature conditions revealed that the correct rates in ALL, Spec+Ceps and Spec+AF conditions were significantly higher than in the Spec and AF conditions (Tukey-Kramer HSD, p<0.05). Three types of SVM optimization functions achieved similar correct rates (Fig. 2C) that were not significantly different in one of the highest dimensionality conditions (ALL condition). In addition, the dimensional reduction of ALL features by the principal component analysis before SVM classification (under L = 7, N = 20 condition) did not improve the classification performance but gradually reduced it with fewer dimensions: 95.4, 98. 6, 99.3, 99.4, 99.5, 99.5 and 99.5% correct classifications obtained for 10, 20, 40, 80, 160 and 320 component conditions, respectively.

### Evaluation 2

**Figure 3:**
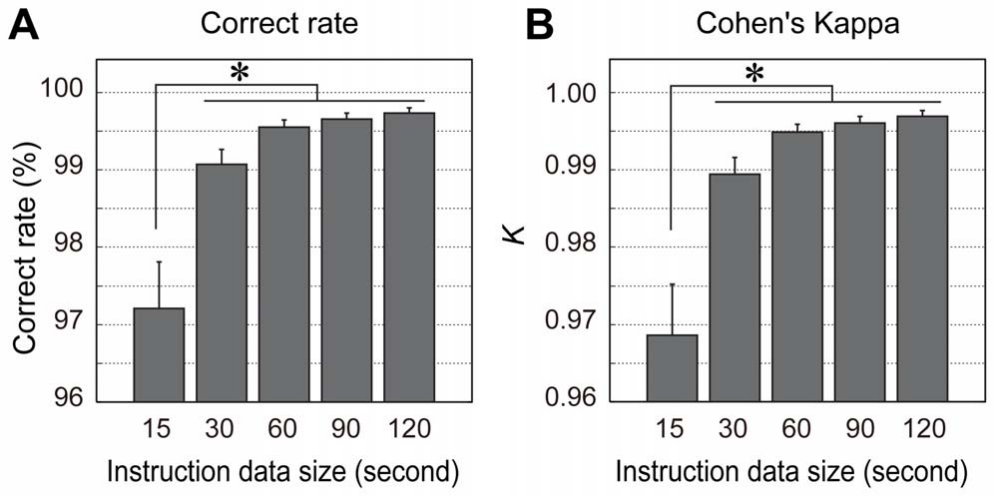
Cross validation performances of two-minute dataset (Evaluation 2). (A) Correct rate percentage for each of instruction data size conditions. (B) Cohen's kappa representing degree of agreement with taking the chance levels into account are calculated for each of instruction data size conditions. Error bars indicate standard error (*n* = 13 birds). *p<0.05 (Tukey-Kramer HSD).

The results of partial cross validation on dataset 2 showed that both the correct rate and Cohen's *Κ* had maximal scores in the 60, 90, and 120 conditions (Fig. 3). The 60-s condition produced sufficiently high performance of 99.5% (±0.33% in SD). Repeated one-way ANOVA demonstrated a significant main effect of the data reducing conditions (correct rate: *F*(4,48) = 16.8, *p*<0.01; Cohen's *Κ*: *F*(4,48) = 17.6, *p*<0.01), and *ad hoc* multiple comparison between conditions showed that scores for both the correct rates and Cohen's *Κ* were significantly lower in the 15-s condition than other conditions (Tukey-Kramer HSD, *p*<0.05).

### Evaluation 3

**Figure 4:**
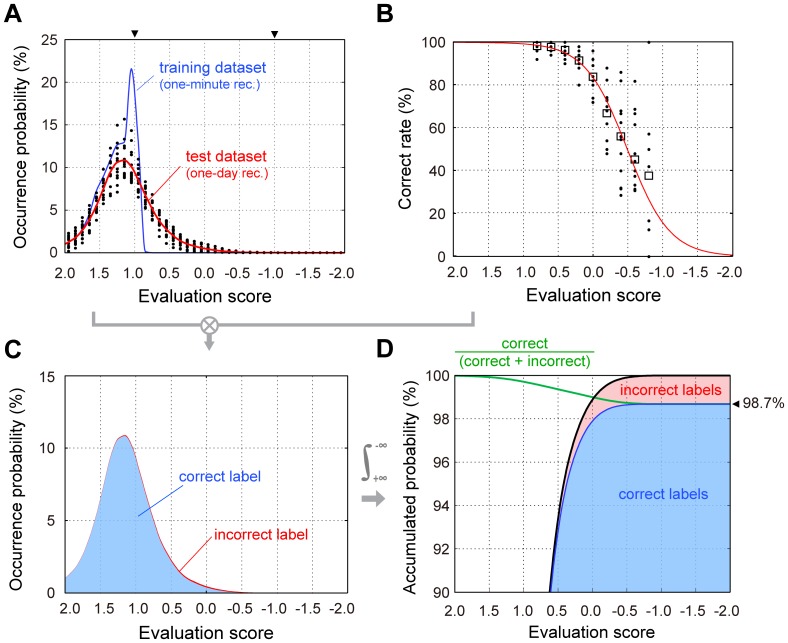
Estimated classification performance for a large dataset (Evaluation 3). (A) Occurrence frequency of evaluation scores (black dots) derived from each bird and a fitted probability density curve (red line) in one-day recording data. Light blue line indicates distribution of evaluation scores of training data (one-minute recording). The evaluation score is an index representing likelihood of its belongingness to any label class, which is normalized as to be ±1 for the classification margins (shown as arrowheads above panel). (B) Correct rate distribution on the evaluation score axis. The correct rates from all birds (black dots) were pooled and averaged (open square), and fitted by a logistic function (red line). (C) Probability density of correct (blue zone) and incorrect (red) were calculated by multiplying the occurrence probability (red line in A; red broken line in C) and the correct and incorrect rates, respectively. (D) Accumulated probabilities of correct (blue area) and incorrect (red area) classifications. The accumulation, or integral, was performed from plus to minus (left to right in figure) on evaluation score axis. Green line indicates an estimated correct rate curve that finally converges to 98.7% (shown as an arrowhead right side of the panel).

The evaluation scores of the large one-day dataset were distributed more broadly in the negative direction (low likelihood) compared to the dataset used for classifier training (Fig. 4A). The subset extracted from lower score locations such as −0.6 or −0.8 contained fewer than fifty samples for some birds. Therefore, we pooled samples from all birds for each location to avoid possible bias from differing sample sizes and performed logistic fitting on the pooled data (Fig. 4B). Then, the correct rate function was multiplied by the occurrence possibility to obtain the occurrence possibility of correct and incorrect classifications (Fig. 4C). Finally, we estimated the overall correct rate for the largest dataset (dataset 3) by accumulating the occurrence possibility of correct labels (Fig. 4D). The estimated correct rate was 98.7%.

## Discussions

### Classification performance

The present study proposed using a linear SVM for semi-automated classification of birdsong syllables, and explored the appropriate size and dimensionality of the instruction dataset and other classifier parameters to design an accurate classifier. Applicability to a large dataset was tested by estimating classification accuracy using a one-day recorded dataset. Cross-validation tests (Evaluation 1) showed that an instruction dataset containing twenty samples per label achieved high accurate classification rates around 99.5%. High classification accuracy using a relatively small instruction dataset in the ALL condition demonstrates the advantages of a linear SVM classifier that generates appropriate discriminant boundaries while avoiding the risk of over-learning with a higher dimensionality feature vector. The results from Evaluation 2 suggest that the data subset corresponding to a one-minute recording could provide sufficient information to cover larger datasets (corresponding to two-minute recordings at least) in a more realistic situation where the occurrence frequency of syllables in the dataset is preserved from the original songs. The final test (Evaluation 3) showed that the estimated performance remained high (98.7%) for the largest dataset (one-day recording). These results suggest that our approach is as efficient as previously proposed methods [3],[4],[5]; however, those methods did not have a mechanism for estimating an optimal training dataset [4]. As previous studies have suggested, using more exemplars in the instruction dataset did not guarantee better performance and sometimes led to more errors [4]. This finding suggests that such methods may require careful selection of training exemplars by trial-and-error. Our experiment quantitatively showed the relationship between classification accuracy and the number of instruction exemplars randomly selected from the larger dataset, which could help minimize the laborious manual labeling process compared to previous methods.

### Reasonable settings for accurate classification

The results from the first evaluation demonstrated that Spec+AF, Ceps+AF, and ALL conditions achieved the highest performance in the cross-validation test. We aimed to include many features to cover the multidimensional variability in song syllables, and the results suggest that higher dimensionality feature space does not impair classification performance. Therefore we concluded that the ALL condition was a reasonable setting. Indeed, for Evaluation 2 performance in the ALL condition (99.55±0.33%) was not significantly lower than the Spec+AF or Ceps+AF condition (99.28±0.49%, 99.54±0.37%) for the 60-s dataset (not shown in figure).

The optimal number of samples requiring manual labeling for an instruction dataset corresponded to approximately one-minute recordings (∼400 syllables) of bouts randomly selected from the entire recording data. Our results suggested that a one-minute dataset contained enough variation to yield correct classification boundaries for larger datasets (two-minute dataset).

Although classification performance did not differ between optimization functions, in practice, there were several reasons to select 2R–2L optimization. In our computational environment (2.8-GHz CPU, 8-GB RAM, Windows 7–64 bit), the times required to train the classifier using the one-minute dataset in the ALL feature space were 61.9±16.4, 61.5±18.7, and 182.4±55.4 ms for the 2R–2L, 2R–1L, and 1R–2L optimizations, respectively (not shown in figure). As demonstrated, 2R–2L and 2R–1L optimizations were faster than 1R–2L. Of course, processing speed depends on the programing language (MATLAB R2012a in our case) and the program library for SVM algorithms. In our environment we opted for 2R–2L (default setting in the program library) as the representative algorithm.

### Remaining misclassification

**Figure 5:**
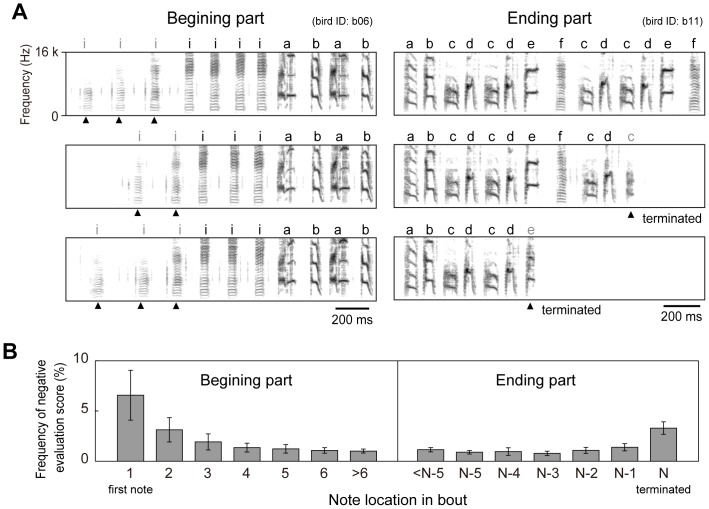
Unstableness of beginning and end of song bout and their evaluation scores. (A) Three spectrograms of beginning (left panels) and ending parts (right) of bouts. Several syllables in beginning parts (shown as arrow heads) of introductory syllables (labeled ‘i’) are often weak and unstable. Last syllables located at the terminated part of bout are sometimes shortened and unclear (shown as arrow heads). (B) Percent occurrence rates of evaluation scores that have negative values, at various syllable locations in beginning (left) and ending parts (right). N indicates the location of terminating syllable. Error bars show standard error (*n* = 13).

The correct classification rate for the large dataset was estimated at 98.7%, indicating that 1.3% of syllables would be misclassified. To investigate the main reasons for such classification errors we analyzed where lower evaluation scores tended to occur within bout, and found that evaluation scores decreased and tended to be negative at the beginning and end of bouts (Fig. 5). These low scores appeared to be caused by acoustical unclearness in the first several introductory syllables of a bout, and deformations of the final syllables. The initial few introductory syllables have been reported to have relatively weaker amplitude and higher spectral entropy (or more noise) in other finch's songs [15], which is consistent with our result showing acoustical instability of those syllables in the Bengalese finch. This finding suggests that classifier performance might improve after eliminating the initial and final syllables prior to classification. Moreover, it is possible to exclude unreliable syllables from classification by thresholding on the evaluation score axis, and label rejected syllables as ‘unknown’. For example, if the rejecting threshold was 0.5, then approximately 94% of all syllables would be labeled with a correct rate of more than 99%, as shown by the estimated result in Fig. 4. Note that the present study regarded the human labeling as correct tutor information without assuming intervention of human errors. Further improvements of song classification could be also introduced with additional error correcting procedure in future studies.

In summary, the present study proposed using a linear SVM classifier for labeling birdsong syllables, in particular, songs of the Bengalese finch as a representative target of neuroethologycal studies. The results showed that an instruction dataset of one-minute recording excerpt (including 387.5±33.1 syllables) could provide sufficient information to classify a larger dataset of one-day long recording (26055.8±17672.3 syllables) with a 98.7% correct rate, indicating that the proposed procedure is suitable for classifying syllables vocalized by one animal model that is generally used in the neuroscientific research field.

## Supporting Information

File S1 Exemplar of two-minutes recording of Bird b09 (WAV format).
(WAV)

File S2 Exemplar of segmented timings and labels for two-minutes recording of Bird b09 (TXT format).
(TXT)

File S3 Entire feature matrices derived from all 13 birds (MATLAB data format).
(MAT)

File S4 Sample script for testing classification (MATLAB script program).
(M) Click here for additional data file.
